# Second toe-to-hand transplantation: A surgical option for hand amputations

**Published:** 2015-06-30

**Authors:** Enrique Vergara-Amador

**Affiliations:** Profesor de Ortopedia y Traumatología. Cirugía de mano y microcirugía. Universidad Nacional de Colombia. Bogotá, Colombia.

**Keywords:** transplantation, toes, microsurgery, hand transplantation

## Abstract

**Background:**

The toe to hand transplantation is a method of reconstruction on the unique or multiple amputations of the fingers. It can be used the whole toe or with certain modifications as a wrap-around flap from the big toe or fingertip. It is a widely accepted option for the thumb.

**Methods:**

It is a series of patients with amputation of one or more fingers of the hand were operated with second toe to hand transplantation. The survival was evaluated and the sensory recovery by 2-point discrimination.

**Results:**

We practiced 12 transplants, 8 thumb, and 4 in other fingers. Ten were adults and two children. All transplants survived. Two patients required tenolysis flexor. The sensibility was recovered with good 2-point discrimination of 8 mm.

**Discussion:**

In the more proximal finger amputations, a second toe is the most appropriate, with lower morbidity of the donor site. The rates of success are between 95 to 100%. We had a success rate of 100%. The resulting defect is in the foot is minimum when the second toe was used. The decision to use one of these techniques depends on the decision and transplant surgeon training. We always used the second toe for transfers to the hand, considering that it will be thinner than the original thumb; our patients had no complaint about the appearance.

**Conclusion:**

Toe-to-hand transplantation is a good technique, providing a very good aesthetic appearance and allowing the recovery of sensitivity. The defect that is created in the foot does not produce significant aesthetic and functional alterations.

## Abstract (es)

**Antecedentes:**

El trasplante de un dedo del pie a la mano es un método de reconstrucción en las amputaciones únicas o múltiples de los dedos. Se puede usar el dedo en su totalidad o con ciertas modificaciones como una envoltura de piel y uña, o de la punta del dedo. Es una opción ampliamente aceptada para el pulgar.

**Métodos:**

Es una serie de pacientes con amputación de uno o más dedos de la mano, intervenidos con un trasplante del segundo dedo del pie a la mano. Se evaluó la supervivencia del trasplante y la recuperación sensitiva con la discriminación de 2 puntos.

**Resultados:**

Se realizaron 12 trasplantes, 8 para el pulgar, y cuatro en otros dedos de la mano. Diez fueron adultos y 2 niños. Todos los trasplantes sobrevivieron. Dos pacientes requirieron tenolisis del flexor. La sensibilidad recuperada fue buena con discriminación de 2 puntos de 8 mm.

**Discusión:**

En las amputaciones más proximales de los dedos, un segundo dedo del pie es el más adecuado, con menor morbilidad de la zona donante. La tasa de éxito está entre el 95 a 100%. Nosotros tuvimos un 100%. El defecto resultante en el pie es mínimo cuando se usa el segundo dedo. La decisión de utilizar una de las técnicas de trasplante depende de la decisión y formación del cirujano.

**Conclusión:**

Es una buena técnica que brinda una apariencia estética muy buena y permite la recuperación de la sensibilidad. El defecto resultante en el pie no produce alteraciones funcionales ni estéticas significativas.

## Introduction

Amputation of the thumb or another finger is devastating for the patient and carries to functional loss, which degree will be depending on which is the amputated finger and the amputation level. The aesthetic defect that occurs causes some problems with regard to their social environment.

The thumb has a few special characteristics that should be evaluated when a reconstruction is performed, such as its position, length, stability, mobility, sensibility and appearance 1-3. The thumb amputation leads to a loss of grip and grasp so that the objectives of the toe to hand transplantation are to provide a new finger capable to make opposition and grip, mobile, that has a good sensibility and improve the aesthetics of patient 1-3.

The concept of the transfer of a toe on hand is attributed to Nicoladoni who around 1899 successfully carried out the transfer of the first toe to the hand in two times, when there was no microsurgery 2. Buncke 4 performed successfully first transposition from the first toe to hand at a monkey, using microsurgical techniques in 1966. As early as 1969, Cobbett reported the technique in humans 5. From these first descriptions, toe to hand transplantation was introduced as a method of reconstruction in the unique or multiple fingers amputations or congenital malformation 6-10. Similarly modifications are designed as partial transfer of tissue, skin and nail wrapping, or fingertip transfers, depending on the need for site receptor 2,3,11-13. Today the toe to hand transplantation has become a widely accepted option in the reconstruction of the thumb, because the role of the opposition.

Instead the toe to hand transplantation is controversial and less accepted when used in finger other than the thumb. However the absence of any finger causes functional limitations and psychological problem, and more when they are multi-finger amputations. Transplanting a toe to the hand, being the finger similar to toe in shape, fingernail, fingertip, etc., is a good alternative to restore function and aesthetic appearance after fingers amputations on the hands.

The aim of this paper is to show the experience with toe to hand transplantation and emphasize the indications in this type of surgery.

## Materials and Methods

This is a case series of patients operated between the years 2003-2013, with one or more finger amputations, who underwent a second toe to hand transplantation. The surgery was performed as a deferred procedure, 10 patients were adults and two children aged 8 and 10 yrs, with a minimum follow-up of 6 months (range 6-34 months).

All patients with absence of a finger trauma were included. Patients whose loss were due to congenital anomalies or did not have a minimum of six months follow up were excluded. All patients remained in the hospital least three days postoperative to pain management and monitoring the transplanted. No measures anticoagulation was used.

### Technical notes

Most surgical procedures for a total or partial transplant takes into account the arterial pedicle going for the first or second toe. It is important to know the arterial tree in this zone 14,15. The first dorsal metatarsal artery is usually the dominant and which is designed transplant. One must know the relationship between this artery with the interosseous muscle and the intermetatarsal ligament. About 30% of cases there are a non-dominant dorsal artery and in this case it should be used the plantar arteries 2,14,15.

Dissection is initiated by the dorsal aspect of the first space, identifying the junction of the lateral digital artery of the big toe and the medial digital artery of the second toe, which arises from the first dorsal metatarsal artery on intermetatarsal ligament 8,16. At this point you can determine the dominance of the dorsal or palmar artery, if the dorsal system is the predominant the connection plantar is tied and dissection of the dorsal artery is continued until to get a good length. If the plantar artery is the dominant, the dissection is continued by the plantar side.

For the same dorsal side it is made the dissection of the venous system, which in general is of good size. A long proximal dissection should be made for an appropriate length. Likewise the extensor tendon of an appropriate length is taken. It is important to take at least one of the digital nerves. For security, it must make a dissection of another artery that could be further used. By the same way long dissection of the flexor tendon is carried out.

First of all mini bone fixation plates or nails is performed, and then proceed with the tendon, the arterial anastomosis, venous and finally the nerve. This order can be varied depending on circumstances.

It is a work with minimal risk. All patients signed an informed consent for surgery and they accepted that information could be included in this article. The identification of the patients was protected.

## Results

**Figure 1.**
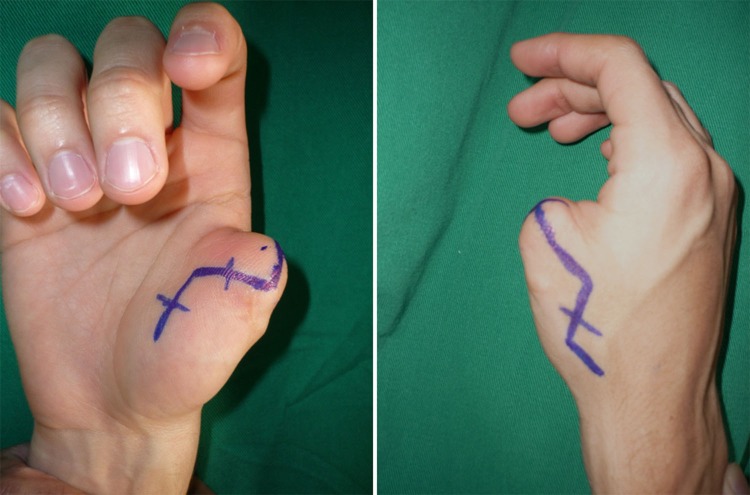
Patient 19 year old, with traumatic amputation of the right thumb of your dominant hand happened two years before the surgery. Incision design is showing.

**Figure 2.**
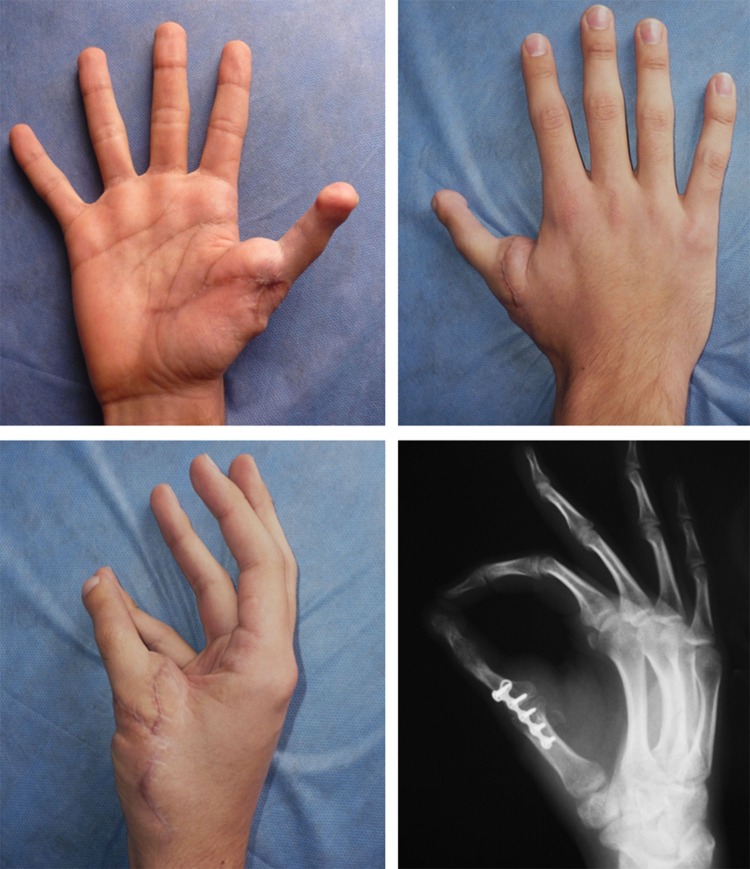
Good result is seen at 8 months at follow-up with the second toe-to-hand transplantation. The patient is happy with the result.

**Figure 3.**
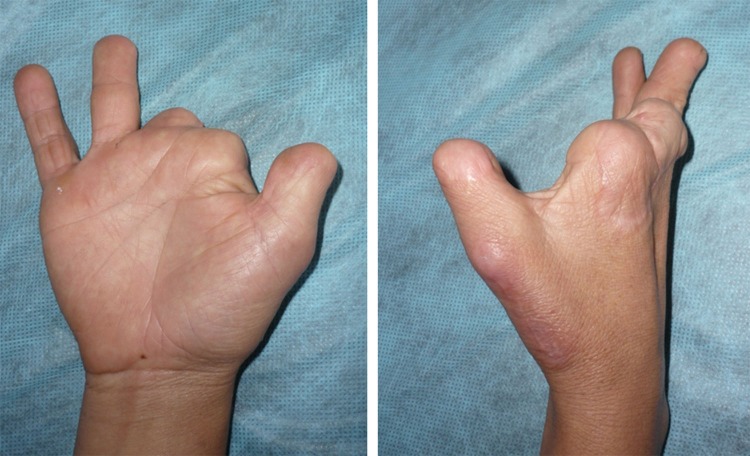
Female patient, 33 year old with partial amputation of thumb and 4th finger, and almost complete amputation of the 2nd and 3rd finger. Previously a lengthening was performed on the thumb. A second toe-to-hand transplantation was indicated.

**Figure 4.**
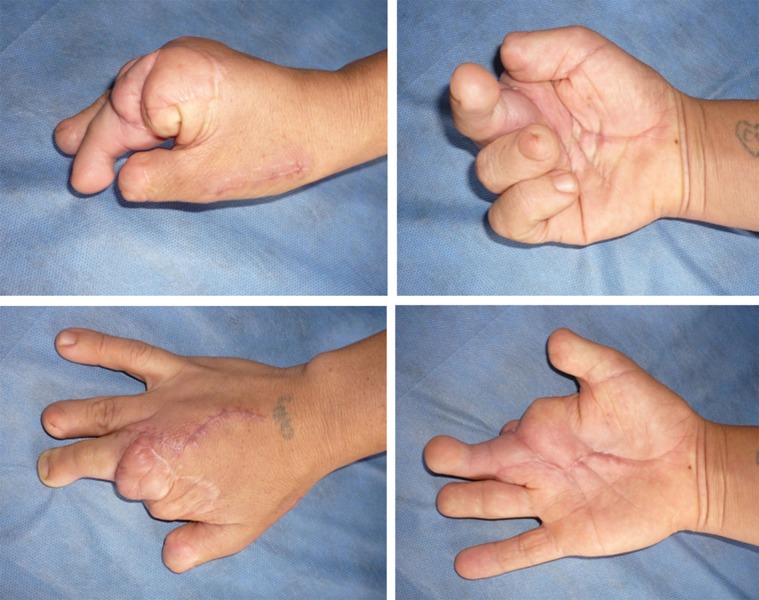
Postoperative result is observed at 4 months after transplantation. She Is very satisfied with their function and aesthetic appearance.

Twelve transplants were made, of which eight were to reconstruct the absent thumb and four to reconstruct other finger different to thumb, two for ring avulsion injuries and two for amputation due to the work machines (Table 1, Figs. 1- 4)

**Table 1.**
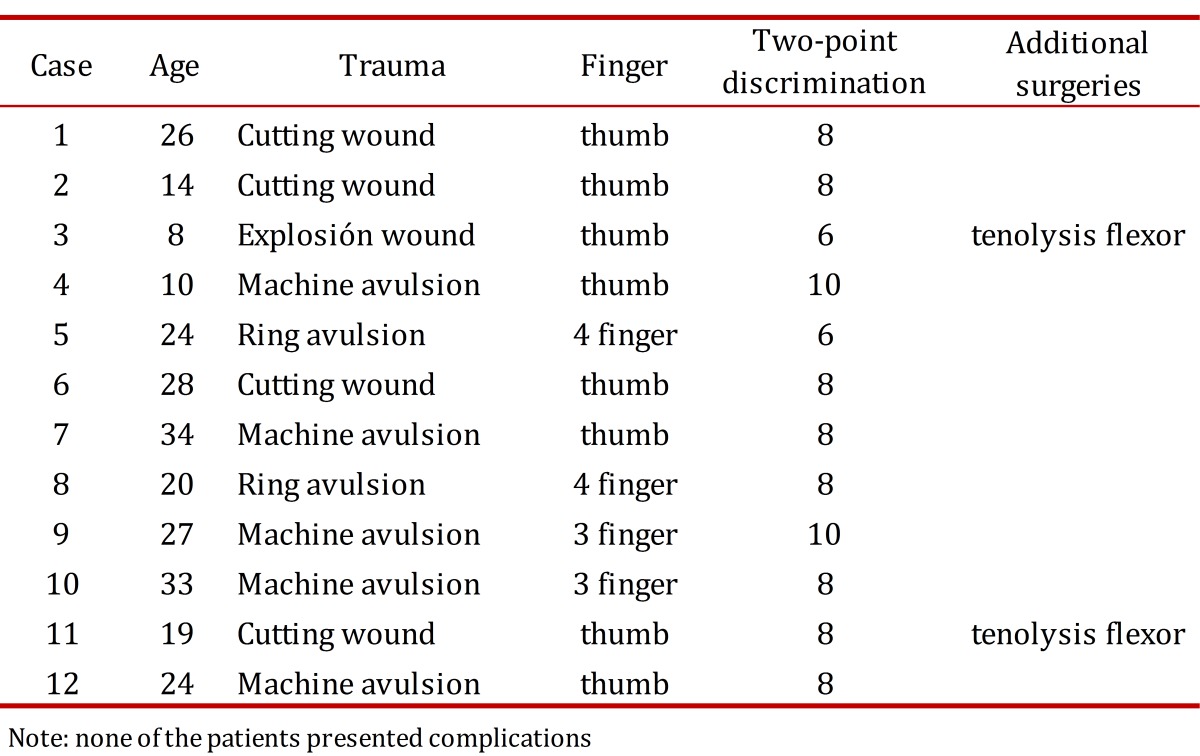
Table of data and outcomes.

All transplants survived without complications. Two patients required tenolysis flexor tendon. Sensibility recovery was good with 2-point discrimination of 8 mm on average (6 to 10 mm).

## Discussion

Toe to hand transplantation is a standardized procedure for reconstruction in traumatic thumb and other fingers amputations 6-13. There are four types of toe transfers to the hand: the second toe, the big toe, the wrapper of the big toe (wrap-around flap) and some specific parts of a toe.

Any of these transfers are preferred when there is a thumb amputation distal to the metacarpophalangeal (MF) joint. However, when the amputation is proximal to the MF joint, a second toe transfer may be the most appropriate option because it allows a bigger exposure of the metatarsal region without increasing the morbidity of the donor site. Any of these techniques have a success rate between 95-100 % 2,3,9,10.

When transferring a toe to the hand is considered an option of reconstructive surgery, the donor site defect should be evaluated with the patient. Although the function of the foot for most activities of daily living is not restricted after resection of one or two toes, there might some limitation for some sports activities.

Aesthetics in the foot should not be overlooked, which could be important for some patients. Consideration should also be taken for some cultural aspects like footwear, or people how tend to be barefoot.

The first toe is bigger and the second toe is smaller than the thumb. There have been some technical devices designed to reduce the size of the first toe so it can match the size of the thumb; also only an envelope transfer can be made (wrap-around flap) 2,3,11-13. On the other hand, the use of the first toe transfer cause a more significant foot deformity compared to a second toe transfer, affecting more significantly the gait.

Although the size of the second toe cannot be increased, and the appearance at the top can be slightly lumped, the defect in the foot is minimal, with aesthetically good results when a careful closure is done.

Particularly, we always used the second toe for transfers to the hand, considering that it will be thinner than the original thumb; our patients had no complaint about the appearance.

If two adjacent fingers need to be rebuilt, the second and third toe can be transplanted in a single surgery 2,3,10. It was proposed to some of our patients, but they did not accept it.

Nowadays, the decision to use one of the toe transfer techniques is based primarily on the taste and the formation of the surgeon. There is no discussion in toe transfer when the thumb needs to be rebuilt, but there is some controversy in the reconstruction of other fingers using these techniques.

In the few cases where we have made it, like in mutilations of several fingers on the same hand, we have had good functional and cosmetic results.

## Conclusion

Toe-to-hand transplantation is a good technique, repairing the loss of a finger with another finger from the same patient, which is the closer possibility to the original amputated finger, providing a very good aesthetic appearance and especially allowing the recovery of sensitivity. On the other hand the defect that is created in the foot does not produce significant aesthetic and functional alterations.
